# The RNA helicase DDX3 and its role in c-MYC driven germinal center-derived B-cell lymphoma

**DOI:** 10.3389/fonc.2023.1148936

**Published:** 2023-03-24

**Authors:** Marion Lacroix, Hugues Beauchemin, Cyrus Khandanpour, Tarik Möröy

**Affiliations:** ^1^ Institut de Recherches Cliniques de Montréal, IRCM, Montréal, QC, Canada; ^2^ Division of Experimental Medicine, McGill University, Montréal, QC, Canada; ^3^ Klinik für Hämatologie und Onkologie, University Hospital Schleswig Holstein, University Lübeck, Lübeck, Germany; ^4^ Département de Microbiologie, Infectiologie et Immunologie, Université de Montréal, Montréal, QC, Canada

**Keywords:** DDX3X, RNA helicase, c-MYC, lymphoma, germinal center

## Abstract

DDX3X is an RNA helicase with many functions in RNA metabolism such as mRNA translation, alternative pre-mRNA splicing and mRNA stability, but also plays a role as a regulator of transcription as well as in the Wnt/beta-catenin- and Nf-κB signaling pathways. The gene encoding DDX3X is located on the X-chromosome, but escapes X-inactivation. Hence females have two active copies and males only one. However, the Y chromosome contains the gene for the male DDX3 homologue, called *DDX3Y*, which has a very high sequence similarity and functional redundancy with DDX3X, but shows a more restricted protein expression pattern than DDX3X. High throughput sequencing of germinal center (GC)-derived B-cell malignancies such as Burkitt Lymphoma (BL) and Diffuse large B-cell lymphoma (DLBCL) samples showed a high frequency of loss-of-function (LOF) mutations in the *DDX3X* gene revealing several features that distinguish this gene from others. First, *DDX3X* mutations occur with high frequency particularly in those GC-derived B-cell lymphomas that also show translocations of the *c-MYC* proto-oncogene, which occurs in almost all BL and a subset of DLBCL. Second, *DDX3X* LOF mutations occur almost exclusively in males and is very rarely found in females. Third, mutations in the male homologue *DDX3Y* have never been found in any type of malignancy. Studies with human primary GC B cells from male donors showed that a loss of *DDX3X* function helps the initial process of B-cell lymphomagenesis by buffering the proteotoxic stress induced by c-MYC activation. However, full lymphomagenesis requires DDX3 activity since an upregulation of DDX3Y expression is invariably found in GC derived B-cell lymphoma with *DDX3X* LOF mutation. Other studies with male transgenic mice that lack *Ddx3x*, but constitutively express activated *c-Myc* transgenes in B cells and are therefore prone to develop B-cell malignancies, also showed upregulation of the DDX3Y protein expression during the process of lymphomagenesis. Since DDX3Y is not expressed in normal human cells, these data suggest that DDX3Y may represent a new cancer cell specific target to develop adjuvant therapies for male patients with BL and DLBCL and LOF mutations in the *DDX3X* gene.

## 1 The DEAD-box RNA helicases DDX3X and DDX3Y

**Figure 1:**
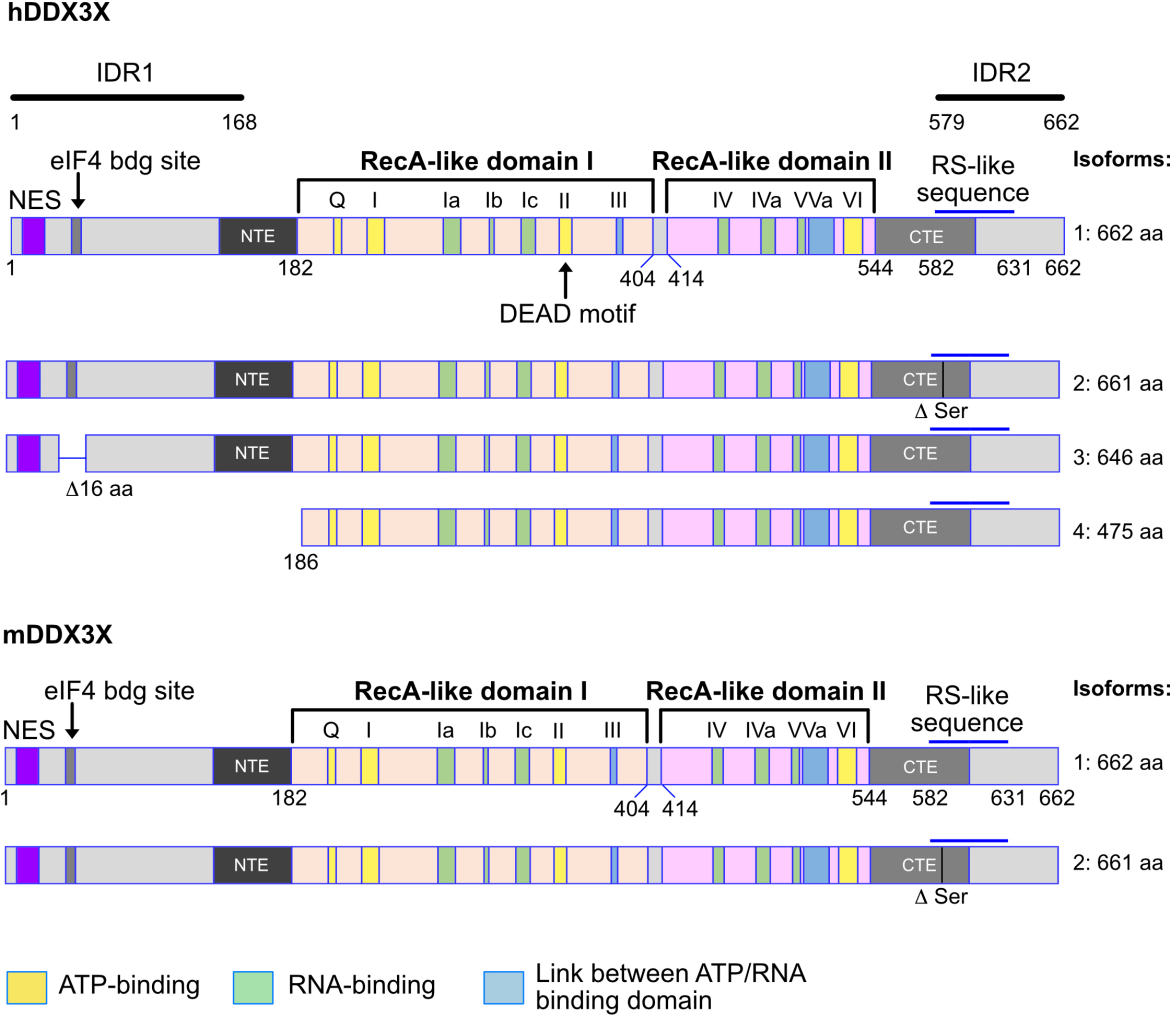
Human and murine DDX3X isoforms. Shown are the different isoforms of the human (upper part) and murine (lower part) DDX3X proteins. The variant 4 isoform of the human DDX3X lacks the 185 N-terminal amino acids, creating a protein that does not contain the nuclear export sequence (NES) and may therefore be localized in the nucleus. This is not the case for the murine DDX3X protein isoform. Above the first variant the structure of the DDX3X protein is indicated as well as the different domains and domain groups. NES, nuclear export signal. RS like sequences: RS-like (arginine/serine-like) region that is responsible for interacting with the nuclear export receptor TAP. NTE, N-terminal extension domain; CTE, C-terminal extension domain. IDR1, IDR2, intrinsically disordered regions 1 and 2. Numbers indicate amino acid residues. See text for details.

DDX3X belongs to the family of DEAD-box RNA helicases, which are named after a common Asp-Glu-Ala-Asp amino acid sequence motif. Eleven additional motifs characterize the DEAD-box family which is exclusively composed of RNA helicases (1, 2) (**Figure 1**). DDX3 is the annotation used to identify two genes named *DDX3X* and *DDX3Y*, respectively localized on the X- and Y-chromosome. *DDX3X* is localized on Xp11.3-11.23 (3) and *DDX3Y* in the AZFa region (4, 5). Because of their genetic localization, DDX3 genes are subject to a sex-specific regulation. The *DDX3X* gene is the X-linked homolog and female mammals (XX) carry two alleles while males (XY) carry only one allele. In females, one of the two X-chromosomes is epigenetically silenced to equalize the dosage of the X-linked gene products between XX females and XY males, a process known as X-chromosome inactivation (XCI) (6–8). XCI causes most X-linked genes to have only one active allele. However, some genes escape from XCI process conserving two active alleles (9, 10). *DDX3X* is as such an escapee in both humans and mice and therefore, females carry two active alleles. The *DDX3Y* gene is the Y-linked homolog and thus, only carried by males. It is not rare for X-linked escapees to have an active Y-linked homolog to suppositively maintain a balanced dosage between both sexes (11, 12). DDX3X and DDX3Y nucleic acid sequences and amino acid sequences, respectively, share 88% and 91% of homology in human (84% and 90% in mouse). Their differences are not equally distributed among their sequences, as approximatively half of their differences are found in their N-terminal domains.

Twelve conserved motifs defining the DEAD box family are found in DDX3X and distributed among the helicase core composed of two RecA-like domains named after the RecA bacterial protein (**Figure 1**), which plays a role in the repair of stalled replication forks, double-strand break repair and general recombination (1, 2, 13–15). The two RecA-like domains are linked by a cleft also named a linker (aa 406-410). The first RecA-like domain contains the following motifs: Q motif (aa 203-207), I (Walker A or P-loop), Ia, Ib, Ic, II (Walker B or DEAD) and III (SAT motif S382). The second RecA-like domains contains the motifs IV, IVa, V, Va and VI. Some of the motifs are thought to be essential for the ATPase activity (Q, I, II, VI), others for RNA-binding capacity (Ia, Ib, Ic, VI), and others for the communication between ATP and RNA binding sites (III, Va). Initially, the minimal functional core of DDX3 was described as the region from aa 168-582 but it was later found to not have any ATPase activity (16–18). Today, the minimal helicase region is defined as the sequence covering aa 182-544 in addition to an N-terminal extension (NTE) and a C-terminal extension (CTE) domains essential for ATP binding and RNA binding capacity (17, 18) (**Figure 1**).

The N-terminal and C-terminal domains contain several motifs and are associated with functions independent of the enzymatic activity. First, there is a known Nuclear Export Sequence (NES) (19–21) in the N-terminal domain. The mutation of the leucine residues 19 or 21 in this region causes an accumulation of DDX3X in the nucleus. Since these leucine residues are conserved in both human and mouse DDX3Y, the NES is very likely present in those proteins too. Secondly, the region from aa 38-43 contains a YXXXXL motif allowing DDX3X to bind to eIF4E (22). This motif is also present in human and murine DDX3Y. Thirdly, a RS-like domain (aa 582-631) was identified in the C-terminal region (23) and has been shown to be essential for the interaction with Tip-associated protein (TAP), a nuclear export receptor (24). In addition, three independent, redundant Nuclear Localization Sequences (NLS) have also been identified: one between the aa 1-139, another one within aa 259-264 and the predicted one between aa 409-572 (20) (**Figure 1**). Also, two intrinsically disordered regions (IDR) exist in the N-terminal (IDR1) and the in the C-terminal part of the protein (IDR2) (19) (**Figure 1**).

DDX3 has a nucleocytoplasmic shuttling capacity (20, 21, 23, 25). DDX3 shuttles to the cytoplasm by binding to TAP through its RS-domain (24, 26) and through the CRM1 binding (21, 25). In the nucleus, DDX3 has been shown to be recruited to the E-cadherin promoter (27) and the IFNγ promoter in the context of an ongoing infection (28). DDX3 can also localize to the centrosome (29) and the mitochondria (30), but no distinction between DDX3X and Y was made in all those studies. However, by comparing both sequences, it is possible that both have similar intracellular localization except when DDX3 localization depends on the N-terminal region after the NES, as X and Y diverge in this region. However, one group generated a DDX3Y specific antibody and has shown that DDX3Y also shuttles between the nucleus and the cytoplasm in human male germ cells (31).

### 1.1 DDX3X isoforms in human and mouse and their functions

**Figure 2:**
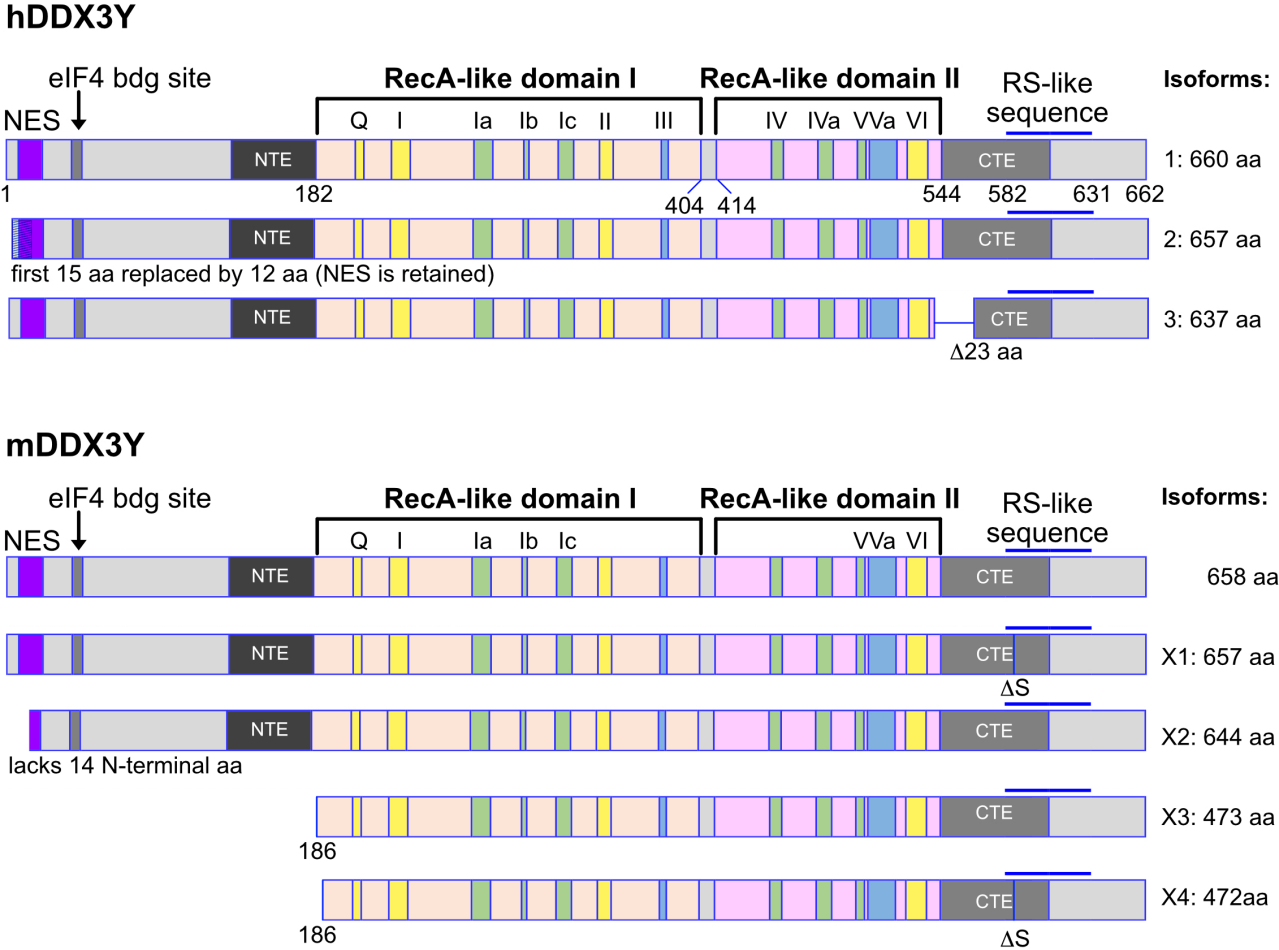
Human and murine DDX3Y isoforms. Shown are the different isoforms of the human (upper part) and murine (lower part) DDX3Y proteins. The inverse situation is found compared to the human and murine DDX3X protein isoforms. Here, the isoforms X2 to X4 of DDX3Y in mouse lack a functional nuclear export sequence (NES). These murine DDX3Y isoforms may therefore also be localized in the nucleus, whereas the human DDX3Y proteins are likely to be mostly cytoplasmic.

*DDX3X* gene encodes different isoforms or variant proteins that are generated by alternative splicing of a pre mRNA transcribed from the same gene locus. Four isoforms (1-4) can be distinguished in human (**Figure 1**). The isoform 4 is shorter than the other isoforms and lacks the N-terminal NES, indicating that this isoform is very likely nuclear whereas isoforms 1-3 could be restricted to the cytoplasm (**Figure 1**). In mice, only one DDX3X isoform containing the NES sequence, this suggests that a nuclear DDX3X is less likely to exist (**Figure 1**). The human and murine *DDX3Y*/*Ddx3y* genes encode three and four isoforms respectively (**Figure 2**). While all three human DDX3Y variants retain their N-terminal NES, the murine DDX3Y isoforms X3 and X4 lack N-terminal amino acids containing the NES and could therefore exhibit a nuclear localization. It remains unknown whether all DDX3X isoforms are present in B cells, where they localize, what are their specific roles and to what extent they are functionally redundant.

The functions of DDX3 proteins are in part defined by their molecular interactions with other proteins and their intracellular localization. A recent comprehensive review has summarized that DDX3X modulates mRNA demethylation and is involved in the transport of HIV transcripts in infected cells (32). In addition, it is a critical factor for translation initiation in general by resolving secondary structures of the 5’-untranslated region in mRNAs during ribosome scanning (24, 33). Other functions of DDX3X include the formation of an anti-apoptosis complex, stress granules assembly and the Tip-associated protein (TAP) dependent nuclear transport of ribonucleoproteins (34). Through the regulating of translation of many other RNAs and through other mechanisms, DDX3 is linked to a variety of cellular processes such as cell cycle progression, apoptosis, DNA damage (35), hypoxia, stress response, WNT/β-catenin signaling, and embryogenesis (34, 36–38). In addition, DDX3 is important for innate immunity through the regulation of the NLRP3 inflammasome, its involvement in the Nf-κB signaling and viral infections reviewed in (32, 39–42). Overall, it remains to be investigated which of these functions can be applied to the B cell context or which of the yet unknown functions of DDX3X is important for B lymphocytes.

### 1.2 Regulation of DDX3Y expression

Male specific *DDX3Y* undergoes a specific translational regulation. Studies suggest that in human *DDX3Y* is widely transcribed but not translated, except in the testis (43). It was shown that a specific structure in the human *DDX3Y* 5’UTR allows its protein expression only in male germ cells (31, 44, 45). In mice, this regulatory process is presumed not to occur (44), and DDX3Y protein expression was found in cardiomyocytes and fibroblasts (46). More recently, DDX3Y protein expression was also found in murine B cells (47). Human DDX3Y was detected in leukemia and lymphoma cells, but undetected in normal B lymphocytes (48, 49).

The DDX3Y protein is very likely ubiquitously expressed in mouse, a main difference with human. However, this difference revealed that loss of DDX3X (*Ddx3x* KO mice) can be partially compensated by DDX3Y. Studies investigating a functional redundancy of both proteins showed that murine DDX3Y and murine D1Pas1, another DEAD-box RNA helicase, can rescue a loss of function of DDX3X in hamster cells (50). Moreover, several other reports have recently provided evidence for a compensatory effect of DDX3Y in DDX3X-deficient mice since male DDX3X-deficient have different phenotypes compared to DDX3X-deficient females, notably in brain cells (51, 52), hepatocytes (53), bone-marrow derived macrophages (54) and hematopoietic cells (47, 55). It was even shown that neurons lacking DDX3X have a higher level of *Ddx3y* mRNA again indicating the existence of a male-specific compensation mechanism, and that both murine DDX3X and DDX3Y have indeed similar or almost identical functions (51, 52). However, a ubiquitous protein expression of DDX3Y in mice similar to DDX3X was never clearly demonstrated because of the lack of DDX3Y-specific antibodies prior to the one we recently generated (47). Consequently, it cannot be fully excluded that, in mice, other male specific factors could compensate for the loss of DDX3X.

### 1.3 Differences between DDX3X and DDX3Y

Studies with constitutive knockout of *Ddx3y* in mouse demonstrated that DDX3Y cannot fully compensate for the loss of DDX3X during embryonic and neuronal development (51). In addition, while the conditional knockout of *Ddx3x* in hematopoietic cells was incompatible with survival for female mice, male mice survived but showed specific deficiencies, for instance in innate antimicrobial immunity or lymphopoiesis, indicating that some functional differences exist between the two proteins in adults (47, 54, 55). The most divergent region between the DDX3X and DDX3Y amino acid sequences is found in their N-terminal part containing the IDR1 (**Figure 1**) (19). Shen and colleagues have demonstrated that this IDR1 is involved in stress granule (SG) formation for both human DDX3X and DDX3Y, but that DDX3Y exhibits a weaker enzymatic activity and thus, promotes less dynamic SGs, causing a higher translational repression compared to DDX3X (19). IDRs are frequently involved in the process of liquid-liquid phase separation (LLPS) driven by weak multivalent interactions. It has been proposed that the divergence between the IDR1 regions of DDX3X and DDX3Y accounts for their different function in LLPS since they share 92% amino acid sequence identity overall. Even if their functions may be redundant (5), it can be expected that functions attributed to DDX3X may also differ from those exerted by DDX3Y, particularly when they involve their N-terminal domains and IDR regions.

The RecA-like domains I and II show more than 96% and 99% sequence similarity between DDX3X and DDX3Y respectively, but all motifs identified within these two domains are completely identical between the two proteins with the exception of the motif Va in the RecA-like domain II, which has a single conservative lysine to arginine substitution (5, 56). The other motifs, namely the RS-like sequence, the CTE and the NTE, all have near 100% sequence similarity and over 80% sequence identity. Only the nuclear export signal (NES) present in both proteins display 80% similarity and only 60% identity. However, despite the differences between DDX3X and -Y, the DDX3Y NES retains its function similarly to that of DDX3X (19). A very similar situation of sequence conservation is observed when comparing murine DDX3X and DDX3Y proteins (57).

## 2 The c-MYC transcription factor and oncoprotein

The transcription factor c-MYC is a nuclear, 62kD phosphoprotein that regulates the expression of many genes involved in cell proliferation, apoptosis, and ribosome biogenesis (58, 59). At its N-terminus, c-MYC has sequences called “MYC box 0-IV” that enable the recruitment of co-factors that mediate its activity as a transcription factor. Typically, these co-factors are enzymes that modify histone tails or regulate the methylation of DNA and thereby directly impact c-MYC target gene expression. At its c-terminus, c-MYC has three motifs comprising a basic region followed by a helix-loop-helix structure and a leucine zipper motif. This “b-HLH-LZ” domain mediates the hetero-dimerization of c-MYC with its shorter partner protein MAX, also a b-HLH-LZ protein, but that lacks a transactivation domain. MYC : MAX and MAX : MAX dimers bind to target gene promoters that contain so-called E-box motifs with a “CACGTG” core sequence. In this situation, the HLH and LZ domains ensure the stability of the c-MYC : MAX or MAX : MAX dimer, while the basic region mediates DNA binding (59, 60).

The critical feature of c-MYC in many human cancers is its hyperactivity caused by chromosomal translocations or amplifications of the *c-MYC* gene leading to increased mRNA expression, or by an enhanced stability caused by mutations or constitutively active intracellular signaling pathways that activate c-MYC expression. An oncogenic activation of MYC can also result from upstream mutated oncogenes such as PI3K or KRAS, which drive MYC expression downstream (61–63). Constitutively active and therefore oncogenic expression of c-MYC has many consequences. It drives cells from G1 into S-phase and thereby enhances cell proliferation. In addition, it is now well established that c-MYC coordinates protein synthesis by directly regulating the expression of ribosomal proteins, the processing of rRNA, the export of ribosomal subunits from the nucleus and the initiation of mRNA translation in such a way that the so-called unfolded protein response (UPR) and other stress response pathways are initiated (58, 64).

### 2.1 The germinal center reaction, c-MYC and DDX3X

One of the pillars of adaptive immunity is the production of specific, high affinity antibodies by B cells and their release into the bloodstream by plasma cells (65–69). This process starts in the bone marrow where lymphoid precursors follow defined steps of differentiation during which the B lymphoid cell fate is determined and the genes for the immunoglobulin heavy and light chains are rearranged by V(D)J recombination (70). This generates a repertoire of mature IgM^+^IgD^+^ B cells that leave the bone marrow and settle in secondary lymphoid organs to form germinal centers (GC) where, upon activation, take place class switch recombination (CSR) and somatic hypermutation (SHM) (71–73), although recent evidence suggests that B cells can undergo CSR before entering the GC (74). Although c-MYC plays an important role in the dark zone of the GC, dark zone B cells undergo somatic hypermutation and proliferate independently of c-MYC (66, 67, 75, 76). Then, dark zone cells enter the light zone, where they are presented with antigens and are selected based on their interaction with follicular dendritic cells and follicular T-helper cells. Cells that do not display sufficiently high antigen affinity for antigen downregulate c-MYC expression and re-enter the dark zone to undergo another round of somatic mutations (65, 68, 77). If a high affinity reaction with an antigen is detected, positive selection occurs, and the cells activate c-MYC expression allowing them to proliferate and expand in size (76, 78–80). However, when cells exit the GC and differentiate into plasma cells, they express BLIMP-1 downregulating MYC expression in later stages of B cell differentiation, most likely at the PB to PC transition (81).

**Figure 3:**
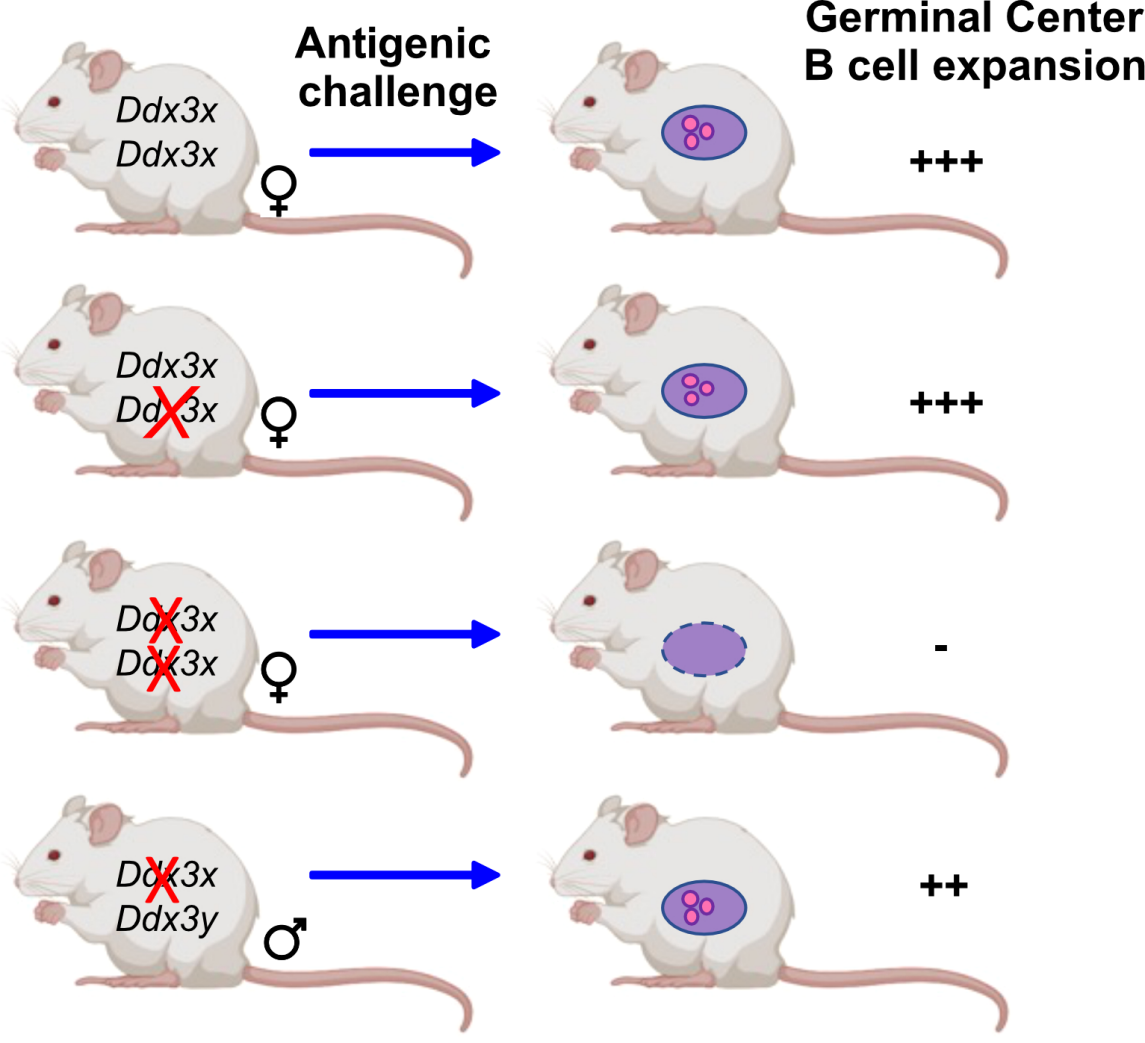
DDX3 activity is required for GC B cells. Mice that carry conditional *Ddx3x* alleles deleted by Cγ1*-cre* upon antigenic challenge show a sex specific reaction: The deletion of one *Ddx3x* allele is well tolerated by female mice and antigenic challenge leads to the expansion of germinal center B cells to the same extent as in wt mice that carry both *Ddx3x* alleles. When both *Ddx3x* alleles are deleted using the Cγ1*-cre* deleter, an antigenic stimulus does no longer elicit the expansion of germinal center B cells. In male mice lacking *Ddx3x* but not *Ddx3y* the development and expansion of germinal center B cells is still possible, but at a lower level compared to wt animals, indicating that DDX3Y can replace DDX3X albeit not as efficiently (47).

DDX3X mRNA is expressed in the germinal center specifically in light zone B cells as shown by single-cell RNA-seq analyses (82) and DDX3X protein is as well present in human and murine mature B cells (47, 48). Two independent studies with several different knockout mouse models showed loss of germinal center B cells upon ablation of *Ddx3x* expression (47, 55) and a reduction of plasmablasts (55). Overall, both studies demonstrate not only that GC B cells are impaired but also show defects several other less mature B cell populations in conditionally deficient mice when *Ddx3x* expression is shut down with the *Vav-cre* or *Cd19-cre* deleter (47, 48, 55). DDX3X deficiency generated with the *Vav-cre* deleter altered hematopoietic progenitors, early B and T cell development and affected marginal zone B cells (47). The deletion of *Ddx3x* using a Cγ1-cre allele, which is inducible by antigenic challenge such as sheep red blood cells, showed that GCs failed to expand in Cγ1-*cre*/*Ddx3* ^fl/Y^ hemizygous males and Cγ1*-cre*/*Ddx3* ^fl/fl^ homozygous females, and that GC B cells were almost entirely eliminated in the absence of DDX3X (47). However, GC B cells were still detected in males, which provided further evidence that DDX3Y can partially compensate for the loss of DDX3X in these cells (**Figure 3**). Although GC B cells were still found in male Cγ1-*cre*/*Ddx3* ^fl/Y^ spleens, immunization with NP-CGG immunization showed that female *Cd19-cre*/*Ddx3* ^fl/fl^ and male *Cd19-cre*/*Ddx3* ^fl/Y^ mice were identically impaired to generate antigen-specific NP+ GC or switched memory B cells (47). Importantly, CSR and SHM that occur in GC B cells require DNA strand breaks that must be repaired without generating an abortive DNA damage response through TP53 activation (83). Studies with *Trp53/Ddx3x* double-knockout mice demonstrated however that TP53 activation is still intact in *Ddx3x*-deficient GC B cells, suggesting that DDX3X probably regulates the proliferative expansion of GC cells and not DNA damage-induced cell death (47).

### 2.2 c-MYC-driven germinal center-derived B-cell lymphoma

Several different non-Hodgkin lymphomas (NHL) can emerge from GC B cells, for example, diffuse large B-cell Lymphomas (DLBCLs), the more indolent Follicular lymphoma (FL) that can progress in rare cases into an aggressive form of DLBCL, or the very aggressive and rapidly growing Burkitt lymphoma (BL) (82, 84, 85). The cells from all three tumor entities contain somatic mutations in their genes coding for the immunoglobulin (*Ig*) variable regions, which is indicative of a GC reaction. Both SHN and CSR require DNA strand breaks, and to allow these processes to happen, DNA damage response checkpoints are either switched off or reduced (71). This situation is by necessity also permissive for off target mutations or chromosomal rearrangements with regions outside the immunoglobulin locus and poses a high risk for GC B cells to undergo malignant transformation. The c-*MYC* gene is a paradigmatic example for these genetic accidents, as two of the hallmarks of GC-derived lymphomas, notably BL, are the activation of c-*MYC* by chromosomal translocation of its coding region to the regulatory elements of the *Ig* locus, and the occurrence of mutations withing the *MYC* coding region that stabilizes the protein by counteracting its ubiquitination and proteasome-mediated degradation (75, 86–88). In the process of lymphomagenesis, c-MYC-overexpressing cells become vulnerable and readily undergo programmed cell death (60, 89, 90). Therefore, cells with secondary mutations eliminating the function of the TP53 tumor suppressor or inducing a gain of function of BCL2 (or related factors) are selected because they counteract c-MYC induced apoptosis and facilitate proliferation and progression to a full blown lymphoma (91). Mutations in these pathways are thus frequently found in c-MYC-driven B-cell lymphomas, as reviewed in (92).

#### 2.2.1 Burkitt’s Lymphoma

Burkitt’s lymphoma is categorized into three types according to histological and immunophenotypic features: the EBV-associated endemic, the sporadic, and the immunodeficiency-associated variant (85, 88, 93, 94). The endemic BL occurs more frequently in children and adolescents in contrast to the sporadic and immunodeficiency-related subtypes; and is characterized by rapid growth and dissemination of tumor cells to liver, kidney, and other organs, including the central nervous system (CNS) (93, 95). Treatment options for BL include standard chemotherapeutic agents (cyclophosphamide, vincristine, methotrexate, doxorubicin, cytarabine, etoposide, prednisone, asparaginase), although some targeted therapies have shown success (e.g., Rituximab) (85, 94–96). When caught early (stage I or II), long-term survival rates are 90% or greater. However, in later stages (III and IV), the survival rates drop to 80-90% and the overall survival is even worse when diagnosed in older patients (97). Of great concern are reports of secondary cancers arising later in life in previously treated young BL patients, most likely due to DNA damage caused by some of the chemotherapeutic agents used. In addition, patients that relapse show great resistance to chemotherapy and remission rates are very low at around 20% (98). There is, therefore, a great need to identify new therapeutic approaches that would reduce the toxicity in BL therapies.

#### 2.2.2 Diffuse large B-cell lymphoma

Diffuse large B-cell lymphoma (DLBCL) is an aggressive non-Hodgkin B-cell lymphoma that shares features with BL but is much more common (99, 100). The therapeutic strategies also resemble those used for the treatment of BL and involve cyclophosphamide, doxorubicin, vincristine, prednisone, and rituximab (R-CHOP). This leads to a cure rate of around 60%, which is lower than that of BL. However, like in BL, patients that relapse are very difficult to treat and have low success rates (101, 102). Transcriptional profiling and mutational analyses were used to categorize DLBCL into subtypes: the germinal center-derived subtype (GCB) and the activated B cell (ABC) subtype with a worse prognosis carrying mutations in the B-cell receptor signaling pathway (103–109). However, more than 10% of patients were neither GCB or ABC and were categorized as unclassified (110).

More recently, a new classification of DLBCL into five major subgroups was proposed based on 574 DLBCL cases analyzed by exome and transcriptome sequencing, array-based DNA copy-number analysis, and targeted amplicon resequencing of 372 genes (106–109). In addition, the occurrence of chromosomal translocations between *Ig* loci and *c-MYC*, *BCL2* or *BCL6* has also been used to stratify DLBCL patients in the clinic using fluorescent *in situ* hybridization (111). In some cases, two or even three translocations occur in a single patient, which has led to the classification of double- or triple-hit lymphoma that all have an invariably dismal prognosis (112). With the advent of whole genome sequencing, DLBCL were re-classified according to their mutational spectrum into six or more subtypes with partially overlapping mutations (113).

Although those lymphomas are commonly treated with chemotherapies; several studies demonstrate that these regimens can be significantly improved. For example, combination of R-CHOP with bortezomib has recently been shown to increase the progression-free survival of DLBCL patients (114). Ibrutinib, inhibiting the Bruton’s tyrosine kinase (BTK) involved in BCR signaling; in combination with R-CHOP also improve event-free survival of DLBCL patients (115, 116). Glofitamab, a monoclonal bi-specific antibody (117, 118), or CAR-T cell therapies may also offer additional options (119). Overall, B lymphoma is still a life-threatening disease requiring alternative treatments. In addition, there are still patients not sensitive to these treatments or have relapsed and are refractory to these treatments, which warrants additional efforts to identify other specific and broadly efficient therapeutic approaches.

## 3 *DDX3X* mutations in Burkitt Lymphoma and diffuse large B-cell lymphoma

In addition to the chromosomal translocations involving the *Ig* heavy and light chain loci and the *c-MYC* gene, somatic mutations in *TP53* and genes coding for proteins involved in the phosphatidylinositol 3-kinase (PI3K) signaling pathway are also frequently found in BL (88, 120–122). More recent efforts using whole genome sequencing and exome sequencing of tumor samples from patients has provided additional and more detailed information on the large spectrum of mutations that occur in BL and DLBCL. Although high-throughput sequencing data have shown that specific patterns and frequencies of somatic mutations exist in these tumors that help to understand their molecular pathogenesis and subtype stratification, many aspects of GC-derived B-cell lymphomas remain to be elucidated. For instance, the reasons for the differences in clinical outcome between pediatric and adult BL patients after initial therapy and poor survival rates at relapse on both pediatric and adult BL remain poorly understood. A recent clinical study comparing adult and pediatric cohorts showed that mutations in the *BCL2* and *YY1AP1* genes are characteristic of adult patients, while mutations in *ID3*, *DDX3X*, *ARID1A* and *SMARCA4* are more frequent in pediatric patients (123). The *DDX3X* gene is one of the most frequently mutated in *c-MYC* translocated pediatric BL after *TP53*, *ID3* and *TCF3* in this study (122–125), but also in others. While the role and mechanistic consequences of loss-of-function (LOF) mutations in *TP53*, *ID3* and *TCF3* have been clarified to a large part, it remains unclear why mutation in the *DDX3X* gene are selected in BL and the question remains whether this protein is indeed a tumor suppressor or not, or whether it exerts both roles in a context-dependent manner (126).

**Figure 4:**
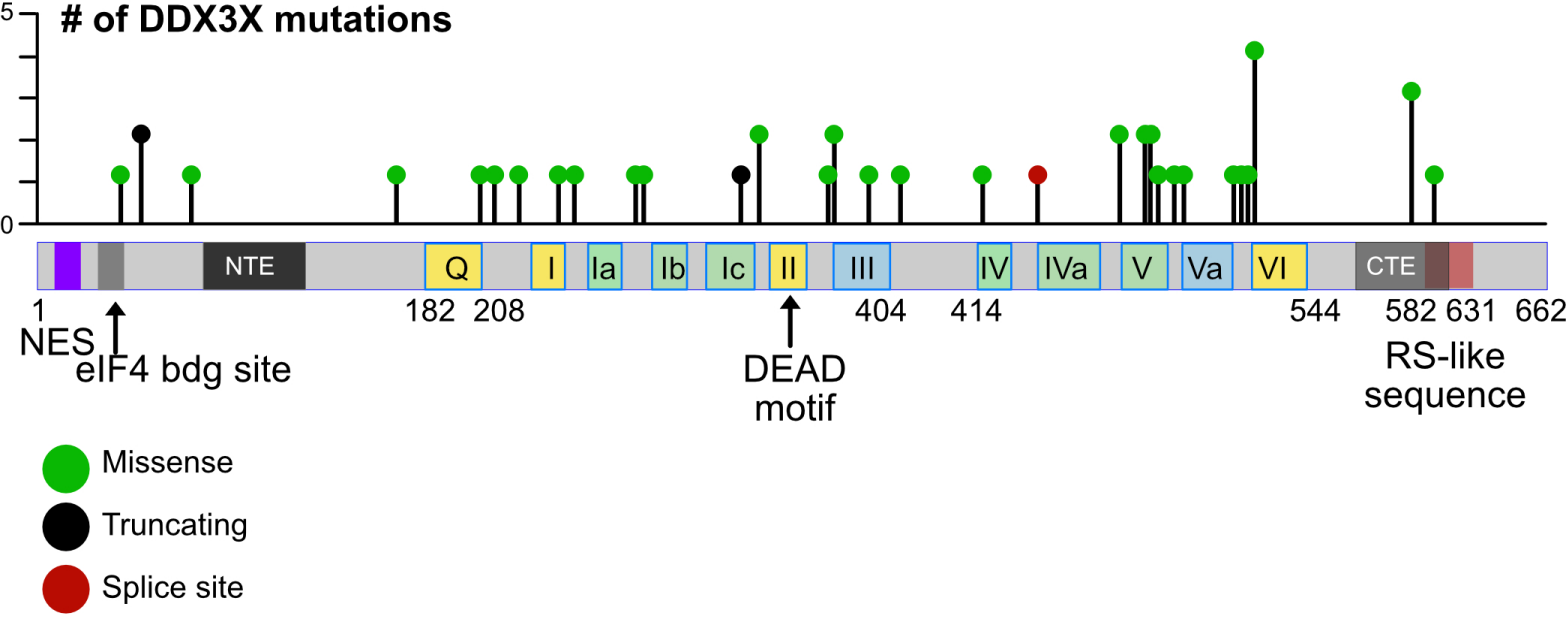
Mutations in the human DDX3X gene. Lollipop plot showing mutations in the DDX3X gene in DLBCL that were identified using cancer-associated genomic database from cBioPortal as an example. The distribution of mutations extends over almost the entire coding region and includes missense and nonsense mutations as well as mutations that introduce splice site errors or truncations, number.

Single as well as double mutations, truncations, frameshift mutations and alterations of splice sites were found in the *DDX3X* gene in BL cells and other hematopoietic cancers, but no specific “hot spot” could be defined (18, 48, 122–124, 127–129) (**Figure 4**). Gong and colleagues have summarized the data from many major cohorts observing that the majority of *DDX3X* mutations found in BL and DLBCL are missense mutations (48). Some of them like the R475 and R534 mutants have also been detected in medulloblastoma and are known to impair the enzymatic activity of DDX3X (17, 18, 130). Similar missense mutants from NKTCL tumors have also shown a decrease of helicase activity in *in vitro* assays (127). These missense mutations detected in BL and DLBCL are characterized as LOF mutations. In addition, a relevant frequency of nonsense mutations and rarer frameshift mutations have been detected in BL and DLBCL (48, 123). Although not clearly demonstrated, they are very likely causing a LOF, particularly when these mutations occur early in the amino acid sequence. Overall, in some cases, large parts of coding sequence are lost; in other cases, changes caused by a mutation appear minimal. It is therefore likely that not every mutation will have the same consequences and it remains to be determined which activity of the protein (i.e., the helicase activity, the ATP binding activity, or the ability to shuttle to the nucleus or other function) is affected by each mutation, and whether these consequences are equivalent in the process of malignant transformation or not. Moreover, whether missense mutations leading to single amino acid changes or truncations at the very end of the protein that spare the helicase domain are also LOF mutants or act as a dominant-negative mutation - or in another yet unknown way - remains be determined. In contrast those missense mutations impairing the helicase activity of the protein are partial LOF mutants (18, 129–131).

### 3.1 *DDX3X* mutations are enriched in MYC-altered GC-derived B-cell lymphoma

It is estimated that 30% of BL harbor a *DDX3X* mutation in addition to the hallmark *c-MYC* translocation making it one of the most frequently mutated gene in this disease (88, 113, 132–136). For BL, three subgroups were identified and named according to the genes that were predominantly mutated: DGG-BL with mutations in *DDX3X*, *GNA13* and *GNAI2*; IC-BL with mutations in *ID3* and *CCND3*; and Q53-BL (quiet *TP53*) in which *TP53* is the only mutated gene besides the *c-MYC* translocations present in all BL cases (113). *DDX3X* is the most mutated gene of the DGG-BL subgroup composed of 85% of BL tumors and 15% of DLBCL tumors. The DGG-BL group that contains the most mutations in *DDX3X* is also the group where mutations in *FOXO1* and *hnRNPu* are enriched and the majority of tumors are EBV+ and harbor evidence for SHM (113). *DDX3X* is also mutated in tumors from the IC-BL subgroup but almost never altered in the quiet *TP53* BL tumors (113). Another study highlights that *DDX3X* mutations are more frequent in pediatric tumors versus adult tumors (123).

*DDX3X* is mutated to a lesser extent in DLBCL (3-5%), although recent analyses have shown a higher mutation rate (around 14%) in DLBCL tumors with altered *MYC* expression (108, 137–139). Thomas and colleagues have observed *DDX3X* mutations in the three DLBCL -A, -B and -C groups. The DLBCL-C group containing *DDX3X* mutations shares some similarities with the DGG-BL group since it includes EBV-positive tumors, with evidence of SHM and a high mutation burden (113). An additional study even estimates that 28% of DLBCL associated with altered *MYC* expression harbor *DDX3X* mutations that are particularly enriched in tumors defined as single-hit lymphoma (*MYC* but no other rearrangements) and c-*MYC* cluster-amplified lymphoma (140, 141). Since it is known that alteration of *MYC* alone cannot induce a lymphoma and rely on other genetic alterations (61, 142), DDX3X is a good candidate to investigate. Studies to classify DLBCL into different subgroups also revealed clearly that LOF mutations in the *DDX3X* gene were enriched in subtypes that carry *c-MYC* translocations over other subcategories of DLBCL, which provides another hint for a link between these two proteins in lymphomagenesis (48, 86, 138). DLBCL patients with *DDX3X* mutation have an inferior 5-year overall survival of 22% compared to 72% for patients with no mutated *DDX3X* allele; although these statistics were assessed independently of the MYC status and inclusion of tumors with MYC translocation in the group of *DDX3X* mutated tumors could have altered the survival (143).

### 3.2 *DDX3X* mutations and sexual dimorphism

*DDX3X* single nucleotide variants and indels were predominantly found in pediatric tumors where 34% of all patient samples harbored at least one *DDX3X* mutation compared to 15% in adult BL cases (113). In the cohort of Lenz and colleagues, the majority were missense mutations, but many non-sense and frameshift mutations were also found (123). Overall, mutations in *DDX3X* occur predominantly in male BL patients with a 46% incidence compared to 4% in females (123). A very strong association between EBV status and a significant overrepresentation of male patients was also observed in the DGG-BL tumors (76% of male patients) and may be the result of a higher *DDX3X* mutation frequency (113, 134, 144). A male bias for *DDX3X* mutations is found in many cohorts as demonstrated by Gong and colleagues (48). Also, distinct sex-specific mutation patterns have emerged, with females having almost exclusively missense mutations and males having mainly truncating mutations in *DDX3X (* 123). *DDX3X* mutations occurring mostly in males could be at least a part of an explanation for the sexual disparity of BL patients.

Given the male predominance in BL with *DDX3X* mutations, the DDX3Y protein has come under scrutiny recently. As outlined above, DDX3Y is not present in human B cells (48) but is found in murine B and T cells (47). A potential explanation for this differential expression between human and mouse could be that mice, in contrast to humans, lack a potentially nuclear isoform of DDX3X missing a NES, and thus may lack DDX3 activity in the nucleus, which could be compensated for by the expression of a nuclear DDX3Y (**Figures 1**, **2**). Whether this is indeed the case has yet to be shown experimentally, but findings that DDX3Y can compensate for the loss of DDX3X in both murine and human cells (145) would support such a model. Although this is possible, other probable explanations exist. For example, the fact that DDX3Y is expressed in murine but not in human B cells may simply be explained by evolutionary processes causing an additional complexity of DDX3Y regulation specific to primates that is not seen in rodents as suggested in the context of spermatogenesis (44). Since *DDX3X* escapes X-inactivation (145, 146), heterozygous LOF mutations are not likely to have a dramatic effect in females since the other allele is expressed and the protein level of DDX3X is not significantly affected when one allele is lost in murine B cells (47). This provides an explanation why *DDX3X* mutations in human B cells are not selected in female GC-derived lymphoma. The situation in male patients is different, as a loss of *DDX3X* is likely to lead to a loss of GC B cells, unless DDX3Y protein is aberrantly expressed (48). This regulation of DDX3X protein expression could be cell type and context specific since other studies have found lower expression levels of DDX3X associated with specific neuronal phenotypes in female *Ddx3x* heterozygous mice (147).

**Figure 5:**
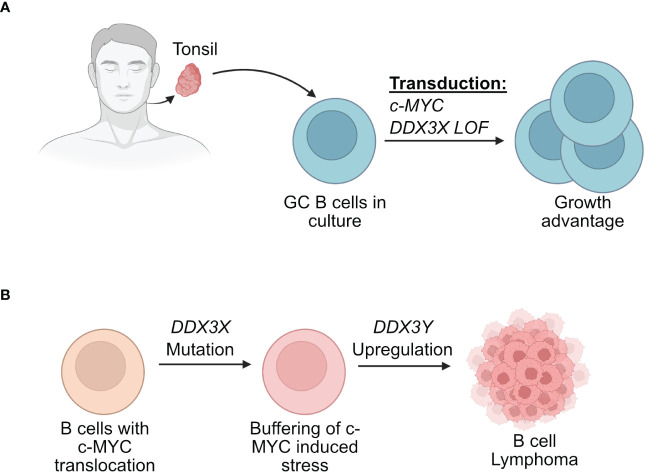
Effect of DDX3X mutations in human B cells. **(A)** Human GC B cells extracted from tonsils have a growth advantage when they are transduced with vectors driving the expression of c-MYC and mutated form of DDX3X. **(B)** Human B cells that acquire a *c-MYC* translocation and a loss of function of DDX3X can overcome c-MYC induced proteotoxic stress but require DDX3Y upregulation for effective lymphomagenesis.

Recent findings from two separate experimental strategies are interesting in this context and may offer further explanations for the sex specific effects on *DDX3X* mutations (47, 48, 148). The first involved human GC B cells from tonsils (48). The use of primary human GC B cells allowed to mimic the GC microenvironment and to model the initial stages of human lymphomagenesis that would have been impossible in established BL cell lines. In this study, primary GC B cells from human tonsils were transduced with retroviral vectors driving the expression of c-MYC and a mutant of DDX3X (48). The co-expression of the mutant DDX3X/c-MYC was able to counteract the cell death-inducing effect of c-MYC overexpression (**Figure 5**). This suggested that *DDX3X* mutations that are found in BL for instance may act in a dominant negative manner since the normal unmutated *DDX3X* was still present in these GC B cells. The hypothesis was put forward that a DDX3X LOF buffers the proteotoxic stress induced by high MYC and allows the cells to proliferate (48).

**Figure 6:**
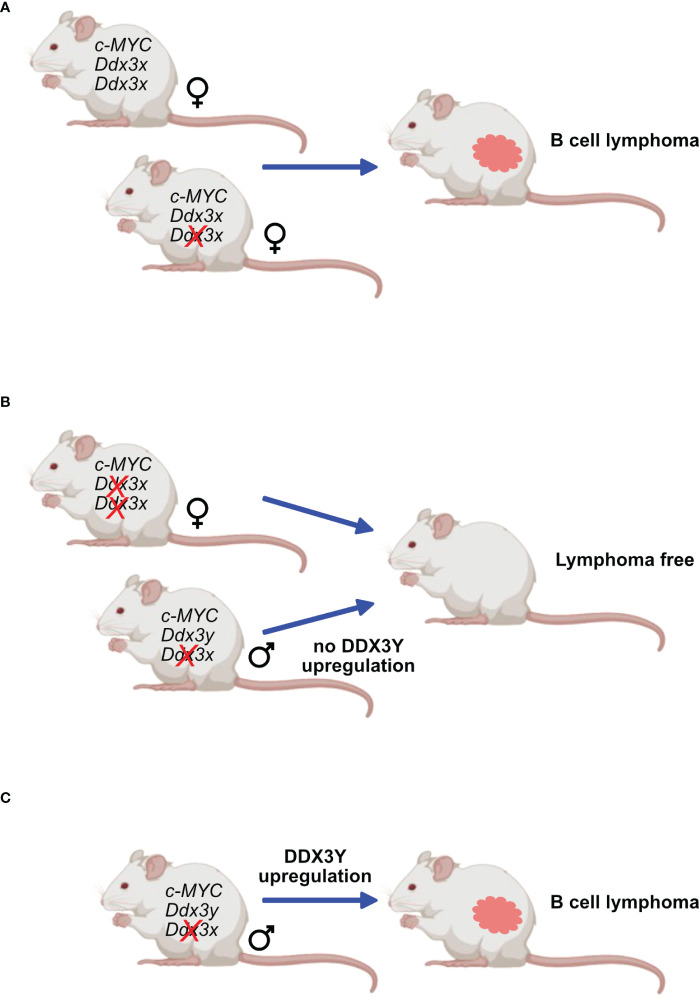
DDX3X and c-MYC in murine lymphomagenesis. Mice expressing transgenes that ensure a constitutive overexpression of *c-Myc* are prone to develop B-cell lymphoma. This process is not affected in females when only one *Ddx3x* allele is deleted **(A)** but is entirely abrogated when both *Ddx3x* alleles are lacking **(B)**. Males deficient for *Ddx3x* that retain the *Ddx3y* allele still develop lymphoma but must upregulate DDX3Y expression **(C)**.

The second approach used transgenic mouse models that are prone to develop B-cell lymphoma owing to a human *c-MYC* transgene either under the regulatory control of the Eµ immunoglobulin heavy chain or Λ light chain enhancer (Eµ-*Myc* and Λ-*Myc* mice) (47). These animals are not perfect models of human BL, since they develop immature or preGC B cell lymphomas rather than malignancies that clearly stem from GC B cells. Eµ-*Myc* mice typically develop pre B cell lymphomas (149) but later stages also IgG^+^ tumors and Λ-*Myc* mice succumb to more mature B cell lymphoma (150) that resemble GC derived since they are B220^+,^ IgD^-^, CD38^lo^ and GL7^+^, but lack CD95 (Moroy et al., unpublished). Although these models do not perfectly recapitulate human BL, they share some features of this disease and have been widely used to study B cell lymphomagenesis. In addition, they were used in this context to assess the effect of *Ddx3x*-deletion in B cells with high MYC expression. In these mouse models, the observations made in human GC B cells were not entirely reproduced and *c-MYC* overexpressing cells that had lost DDX3X did not expand and only those lymphomas were obtained that did not delete *Ddx3x*, indicating that loss of DDX3 activity is incompatible with *c-Myc* induced lymphomagenesis in mice (47) (**Figure 6**). This difference may however be explained by the fact that a *Ddx3x* deletion may act differently compared to a LOF mutation.

However, one observation that remained coherent between the two studies is that both models, male BL patients and male mice with immature B cell lymphoma, show respectively aberrant and high DDX3Y expression that compensates for the loss of DDX3X (47, 48) (**Figures 5**, **6**). Altogether, these two studies confirmed a primordial compensatory role of DDX3Y in B cells and clearly indicated that sex-dependent differences must exist in animals and patients with *DDX3X* LOF mutations with consequences for the process of B cell malignant transformation.

### 3.3 The link between c-MYC and DDX3X in GC- derived B-cell lymphoma

If *DDX3X* is required for GC B cells and GC B cell-derived lymphomagenesis, why are mutations in this gene selected for in human BL or in *c-MYC* altered DLBCL? This question remains to be fully resolved, but the model that was proposed in the experiments with human B cells mimicking GC B cells posits that given the role of DDX3X in ribosome biogenesis and mRNA translation, a *DDX3X* LOF mutation may facilitates the early stages of *c-MYC*-driven lymphomagenesis in males by moderating the increase in global protein synthesis caused by MYC overexpression, thus ensuring cell survival (48, 148). It would be only once a lymphoma is established that aberrant expression of DDX3Y can be induced in malignant B cells to restore full translation capacity and to adopt a level of protein synthesis required for tumor development. However, *DDX3X* LOF mutations are frequent in human BL but never seen in murine B-cell lymphoma modeling BL (48). This can be explained by the fact that lymphoma develop from less mature B cells in the Eµ-*Myc* and Λ-*Myc* models and not from fully mature GC B cells. This supports a GC-specific cooperation between *DDX3X* mutations and *MYC* translocation. Moreover, the hypothesis put forward by Gong et al. cannot explain data that haploinsufficiency for Rpl24, which reduces global protein synthesis, abrogates *c-MYC*-induced lymphoma (151). Also, the *DDX3X* mutations collaborate only with activated c-MYC and not with high level of expression of BCL2 or BCL6 (48), suggesting that DDX3X possesses functions that specifically help cells to maintain a high level of MYC. Because mouse models in which one or two alleles of *DDX3X* can be deleted do not reproduce the phenotype seen with human B cells that have acquired mutations leading to a partial LOF or a dominant negative allele, further experiments with mice that harbor conditional knock-in alleles allowing expression of human *DDX3X* mutant alleles could be informative as to why DDX3X/Y requirement for B-cell lymphoma diverge between mice and humans.

## 4 DDX3X mutations, therapy resistance and disease outcome

Comparatively few studies addressed the question whether mutations in the *DDX3X* gene correlate with therapy resistance, relapse frequency or were indicative of disease outcome. One study analyzed DLBCL patients with *DDX3X* mutations and found a significantly worse median overall survival of somewhat over 40 months compared to cases with unmutated *DDX3X* (> 200 months) (143). The 5-year overall survival of DLBCL patients with *DDX3X* mutations was found to be only 22% compared to 72% for patients with an unmutated *DDX3X* gene. However, the authors mention that the availability of the data for progression-free survival or event-free survival was insufficient to draw firm conclusions or to assess the survival independently of MYC status (143). The only other data set on survival of patients with hematopoietic malignancies come from a study with NK-T cell lymphoma patients, which have a different pathogenesis than the GC derived NHLs discussed here (127). However, like DLBCL, the disease outcome of NK-T cell lymphoma patients is worse when mutations in the *DDX3X* gene are present. *DDX3X* was even associated with worse overall response and progress free survival in lenalidomide-refractory chronic lymphocytic leukemia patients (152).

Experiments with cell lines that constitutively expressed a DDX3X-R475C mutant generated using the Crispr/Cas9 technology or that expressed lower levels of DDX3X through shRNA- and siRNA-mediated knockdown led to resistance to doxorubicin and to HDAC inhibitors (143), suggesting that *DDX3X* mutations are linked to chemoresistance observed in the clinic, although the molecular basis for this remains to be elucidated. The same study also found that cell lines with *DDX3X* mutations or *DDX3X* knockdown enhances the proliferation and their migratory potential of lymphoma cells lines, whereas overexpression of an unmutated DDX3X decreased proliferation. This data concurs in some regard with findings from Hodson and colleagues, who saw a competitive growth advantage in tonsil cells transduced with *c-MYC* and the dominant-negative DDX3X mutant that did not observe this competitive advantage when cells were transduced with *c-MYC* and the unmutated DDX3X (48).

## 5 Therapeutic potential of targeting DDX3X and/or DDX3Y

**Figure 7:**
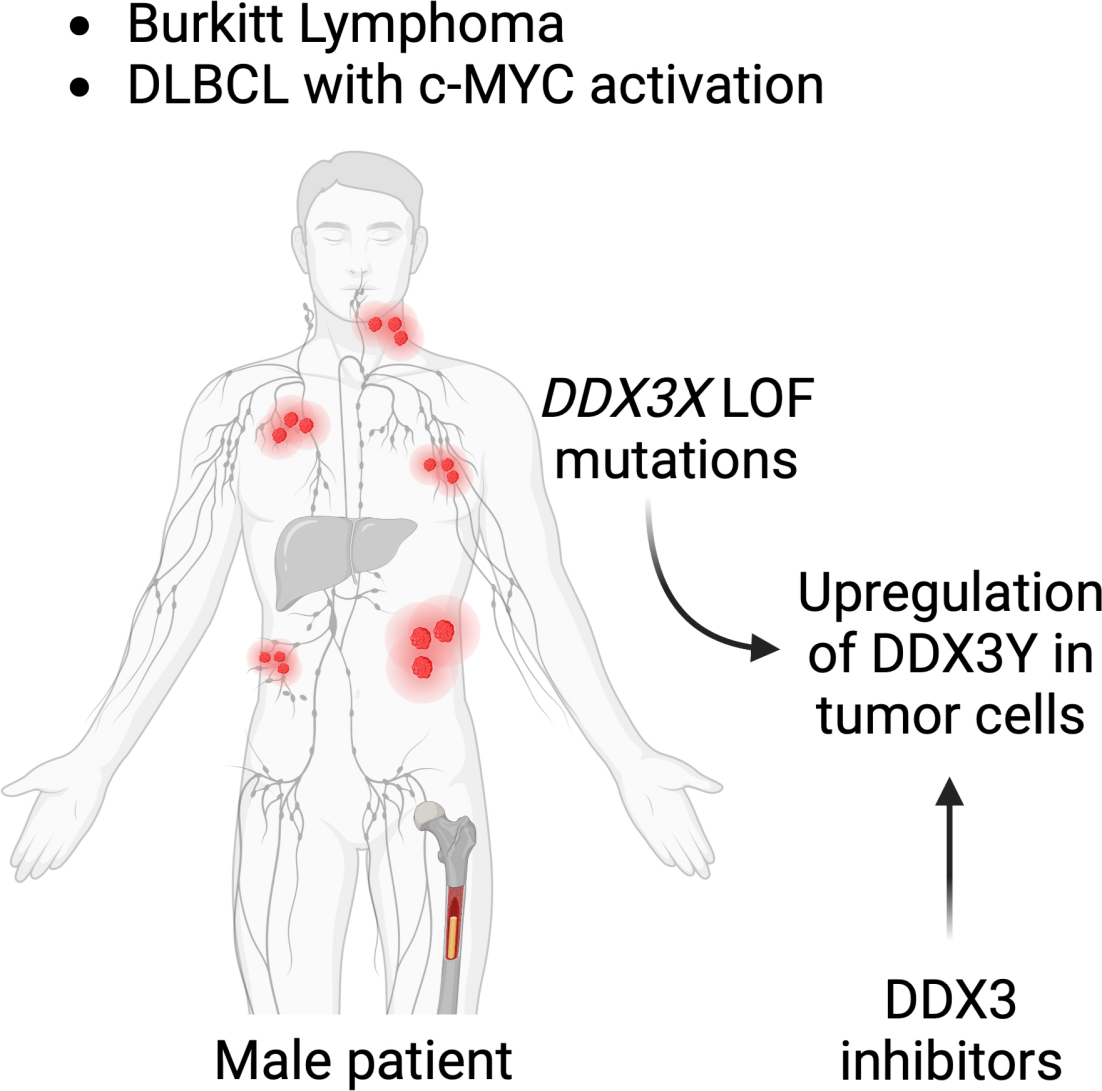
DDX3 as a target for lymphoma treatment. Male patients suffering from BL or subtypes of DLBCL with c-MYC activation who have mutations in their *DDX3X* gene, may be treated with DDX3 inhibitors. DDX3Y is not expressed in other human cells and DDX3Y aberrant protein expression specifically seen in lymphoma cell could provide a tumor type specific target.

With all their disparities and differences, both the human and murine models demonstrate a requirement of DDX3X or DDX3Y activity for c-MYC driven B-cell lymphomagenesis making them attractive therapeutic targets (153). DDX3 being an essential gene, at least one of the two paralogs is indispensable for cell survival (154, 155), particularly in a B cell lymphoma context as shown by the aberrant DDX3Y expression in human lymphoma with a DDX3X LOF; and by the DDX3Y overexpression in murine lymphoma cells with a *Ddx3x* KO (47, 48). Although this needs to be experimentally demonstrated in the context of a B cell lymphoma, it suggests that targeting the intact paralog in those lymphoma cases that harbor a c-MYC activation would eliminate cancer cells and represent a rather specific therapeutic strategy. Moreover, given that a pharmacological inhibition of c-MYC is difficult (156), inhibition of DDX3X or DDX3Y could represent alternative therapeutic avenues for the treatment B-cell lymphoma in male patients with *DDX3X* LOF mutations (**Figure 7**).

### 5.1 DDX3 enzymatic inhibitors

Since DDX3X and DDX3Y are enzymes with an ATP binding site and an enzymatic helicase domain, small molecule inhibitors can be designed and tested. To date around 20 of such inhibitors that target either the ATPase or the helicase activities of DDX3X have been generated by different groups and have been tested *in vitro*, and in a few instances also *in vivo* in xenograft models for glioma or breast cancer (153). However, none of these inhibitors were yet evaluated for their therapeutic suitability for B-cell malignancies such as BL or DLBCL. Although it has not been formally demonstrated, these inhibitors likely inhibit both DDX3X and DDX3Y, given that the amino acid sequences of both the ATPase and helicase domains are almost identical (19, 157). Furthermore, it is not unlikely some of the inhibitors also affect other DEAD-box helicases since their enzymatic domains are highly conserved between them.

One strategy to find a specific inhibitor could be to target a structural entity within the helicase domain that is unique to DDX3X or DDX3Y. Although highly conserved in all DEAD-box helicases, DDX3X and DDX3Y helicase core domains contain a unique 10 aa insertion (250-259), between the motifs I and Ia, referred to as “DDX3 insertion” (16), which is also found in the murine DDX3X (158). It was presumed important for RNA binding (16) and it was shown later that a deletion of this sequence in the human DDX3X homologue induces a reduction in ATPase and unwinding activity (158). The exact function of this sequence is unclear, but it is highly interesting for drug design because this sequence is specific to DDX3 and not to other closely related DEAD-box helicases; it is important for enzymatic activity and is slightly differing between the X and Y homologues.

Testing DDX3 inhibitors for treating patients regardless of sex or mutational status may be efficient but also challenging because of the toxicity associated with *Ddx3x* deficiency (47). Indeed, systemic and complete ablation of DDX3X in adult mice has toxic side effects, probably owing to the destruction of intestinal epithelial cells leading to an inflammatory phenotype with features typical of septic shock (Moroy et al., unpublished). Red blood cells are particularly sensitive to *Ddx3x* deletion suggesting that anemia could be a consequence of DDX3X inhibition (47). These effects have been observed in female KO mice highlighting the potential damages of using inhibitors in female patients or male patients without any *DDX3X* mutation (since DDX3Y is not expressed in this case). Under these circumstances, a potential future clinical application of any DDX3X inhibitor has therefore to be carefully monitored and tested with doses that remain too low to cause these side effects.

However, patients with a loss of DDX3Y – i.e male patients whose cancer cells have lost their Y chromosome – carry lymphoma cells relying only on the DDX3X paralog (155). Consequently, this subset of patients may be more sensitive to DDX3X inhibitors and could be treated with lower doses thus limiting toxicity. It is also likely that DDX3X inhibitors would be more effective in combination with other established lymphoma drugs such as cyclophosphamide, etoposide, doxorubicine and others, allowing to reduce their dosage and thereby overall toxicity and enhancing their effectiveness. Conversely, in lymphoma patients harboring a *DDX3X* mutation, selective inhibition of DDX3Y without affecting DDX3X would be extremely advantageous as it would target only lymphoma cells relying on DDX3Y. This strategy would spare normal cells that do not express DDX3Y and then offer a very specific option.

### 5.2 Targeting DDX3Y independently of its enzymatic activity

Due to its very restricted expression, targeting DDX3Y in lymphoma patients harboring an activated *c-MYC* allele in addition to a DDX3X LOF is very likely the best strategy. As an alternative to targeting DDX3Y enzymatic activity, a direct or indirect elimination of the DDX3Y protein may be sufficient to eliminate cancer cells (154). New approaches with antisense oligos or PROTAC technologies may be among the strategies for such an approach (153).

## 6 Conclusion

The *c-MYC* oncogene is activated in almost all human cancers and is most likely the critical driver in most if not all GC derived B-cell lymphoma. However, activation of *c-MYC* alone does not achieve a full malignant transformation and an effective process of lymphomagenesis requires additional mutations. One piece of evidence for this concept comes from the studies of c-MYC transgenic mice that develop monoclonal lymphoma originating from a particular individual cell and not from all cells that express the transgene. Other experimental findings that support the notion that c-MYC requires cooperating events are the results from sequencing of BL or c-MYC altered DLBCL that show mutations in *TP53, ID3, TCF3* or *DDX3X* and other genes. Among these cooperating events the LOF mutants of DDX3X stand out because it is less clear how they act in concert with c-MYC. In contrast to other mutations, for example in *TP53*, which accelerate lymphomagenesis in a c-MYC transgenic mouse model when deleted, the complete abrogation of DDX3X is incompatible with the emergence of B-cell lymphoma, excluding a role as a tumor suppressor at least in mice, whereas experiments with human GC cells show the opposite. This makes a modeling of human BL in the mouse difficult but that could potentially be remedied by the engineering of a conditionally deficient *Ddx3y* allele with the concomitant conditional knock in of a *DDX3X* mutation.

Given its critical role in so many human cancers, c-MYC is at the center of attention as a potential therapeutic target, but its lack of enzymatic activity has made this task extremely difficult. For instance, it has been attempted to interrupt the MYC : MAX complex by small molecules, but poor bioavailability and lack of selectivity has limited their use. Omomyc, a dominant negative MYC peptide which can interfere with MYC in malignant cells has shown promise and is presently in clinical trials. However, Omomyc is a peptide over 80 aa long and its suitability and usefulness as a drug in the clinic has still to be demonstrated. It remains therefore necessary to find other ways to broaden the repertoire of molecules to target the functions of c-MYC in tumor cells. Since MYC hyperactivation alone does not induce lymphomagenesis, genes that are often found co-mutated in situations of MYC translocation are of interest as potential therapeutic targets.

The recent discovery of the aberrant expression of DDX3Y compensating the loss of DDX3X function in tumors with *c-MYC* activation has opened a new area for developing new therapeutic strategies for B cell lymphomas. Indeed, the situation of BL patients and the c-MYC-altered subset of DLBCL patients harboring DDX3X LOF mutations may render the use of DDX3 inhibitors beneficial for this selected group of patients. Since these patients are almost exclusively males, and their lymphoma cells rely on the aberrant expression of the DDX3Y protein, DDX3 inhibitors could be beneficial for eliminating cancer cells. In this case, specific DDX3Y inhibitors blocking its enzymatic activity or any other strategy abolishing DDX3Y protein would eliminate the B-cell lymphoma, but not other cells, since DDX3Y is not expressed in most normal cells. Therefore, toxicity would be limited, and the treatment would be specific to cancer cells. In addition, the experiments with lymphoma-prone transgenic mouse models showed that malignant B cells with a c-MYC activation are highly sensitive to loss to DDX3, suggesting a synthetic lethality between gain of MYC and loss of DDX3 function that could be exploited by small molecule inhibitors. The main challenge with this concept remains the high similarity between DDX3X and DDX3Y, but if a strategy could be devised in which DDX3Y could be targeted over DDX3X, the benefits for male BL and DLBCL patients with DDX3X LOF mutations would be significant and could give rise to the development of a novel cancer cell-specific therapy.

## Author contributions

ML, HB, and TM wrote the main text. TM and HB made Figures. CK wrote text pertaining to clinical data and GC derived lymphoma. All authors contributed to the article and approved the submitted version.
